# The swift study: dietary profiles of night shift workers characterised by overweight and obesity

**DOI:** 10.1007/s00394-026-03917-9

**Published:** 2026-04-10

**Authors:** Angela B. Clark, Alison M. Coates, Corinne Davis, Gloria K. W. Leung, Yan Yin Phoi, Michelle Rogers, Rochelle Davis, Maxine P. Bonham

**Affiliations:** 1 Department of Nutrition, Dietetics and Food, Monash University, 264 Ferntree Gully Road, Notting Hill, Melbourne, VIC 3168 Australia; 2 Department of Allied Health, Swinburne University of Technology, Hawthorn, VIC Australia; 3 Alliance for Research in Exercise, Nutrition and Activity (ARENA), Adelaide University, Adelaide, Australia; 4 Behaviour-Brain-Body Research Centre, Adelaide University, Adelaide, Australia

**Keywords:** Shift work, Diet, Metabolic health, Overweight, Obesity

## Abstract

**Purpose:**

This study aimed to explore dietary profiles of a night shift working population with overweight/obesity and the impact of different shift work schedules (rotating vs. fixed night) on their diet.

**Methods:**

Participants from a randomised controlled trial (SWIFt trial) provided 7-day food diaries at study enrolment. Mean energy intakes (EI), and mean percentage of EI as nutrients (macronutrients, saturated fat, added sugar, alcohol) grams of fibre and milligrams of sodium, were assessed against recommendations for Australian adults. Energy intake was assessed for underreporting. Regression analyses were performed on nutrient intakes whilst controlling for individual and lifestyle factors (age, gender, BMI, physical activity, shift work exposure, occupation and shift schedule).

**Results:**

The diet of night shift workers (*N* = 245, aged 46.8 ± 9.8 (mean ± SD) years and 53% female) was characterised as high in fat, saturated fat and sodium, while low in carbohydrate and fibre compared with recommendations, regardless of shift work schedule. However, the mean 24 h EI of rotating shift workers was higher than fixed night workers (9329 ± 2915 kJ/day vs. 8025 ± 2383 kJ/day, *p*< 0.001), even after assessing plausible EI reporters only (*n* = 130) (10968 ± 2411 kJ/day vs. 9307 ± 2070 kJ/day, *p*< 0.001). Furthermore, those performing rotating schedules presented with higher sodium and alcohol consumption.

**Conclusion:**

These findings support the evidence of shift work being associated with poorer dietary profiles, which likely contribute towards worsened metabolic health amongst night shift workers. Dietary strategies that target problematic eating habits and address unique challenges of working shift schedules are needed to mitigate metabolic health risk in shift working populations.

**Supplementary Information:**

The online version contains supplementary material available at 10.1007/s00394-026-03917-9.

## Introduction

In Australia, approximately 16% of the working population are shift workers [1], that is, they perform a work schedule at different hours to the standard “work day” hours performed between 6am to 6 pm [2]. Nearly half (45%) of Australian shift workers follow a rotating pattern [3] entailing regularly changing shifts that require workers to switch between day and night work, while their work schedules may also vary in direction (clockwise or counter clockwise) and speed (fortnightly, weekly or rapid) [2]. Unlike fixed or “permanent” night shift schedules, which set out unchanging and consecutive night shifts and days off, rotating shifts may include a variation of consecutive morning, day, afternoon and night shifts followed by days off [2]. Industries such as transport and health, rely on workers for 24-h business operations, however it is well established that shift work is associated with an increased risk of overweight, obesity [4] and chronic diseases such as cardiovascular disease (CVD) and type 2 diabetes (T2D) [5, 6].

Reported dietary trends of shift workers, such as high intake of snacks and discretionary foods and drinks [7, 8], irregular meals [9] and a greater distribution of energy intake overnight compared to the day [10, 11] are known to negatively impact health. Eating at night is associated with increased metabolic health risk [12–15], as humans are instead primed to sleep during the “biological night” from ~ 2100 to 0700 h, when melatonin is secreted at high concentrations [16]. Consuming a greater proportion of energy at night increases the risk of insulin resistance, and lowers glucose and lipid tolerance [17–19], which are each factors associated with risk of T2D. When 24-h dietary energy intake is similar but redistributed to night-time hours on account of night shift work [18], or an identical meal eaten during the morning (ie. 0800 h) is consumed in the evening (at 2000 h) [17], studies observe a time of day effect to impaired post prandial glucose and lipid control with night time eating. Furthermore, eating at night may promote weight gain as energy expenditure is lower due to a reduced metabolic rate driven by the circadian phase of sleeping and fasting [20, 21].

The contribution of shift schedule to dietary intake may also be a factor for increased metabolic health risk. A recent scoping review reported that macronutrient intake and foods consumed by shift workers are impacted by shift schedule and work industry [22], and a recent systematic review and meta-analysis observed high energy intakes in rotating shift workers which over time, may contribute to weight gain [23], suggesting the need to explore the influence of work-related factors on shift workers’ diets.

Dietary habits of shift workers are likely influenced and altered by practical demands and challenges associated with fixed or rotating schedules, that impact on eating habits such as meal timing and fasting windows. Nurses with rotating schedules report more irregular eating patterns, higher food consumption and more late night snacking than their counterparts undertaking fixed day or night shift work [9]. Rotating shift workers in the mining industry report great variation in timing of meals across shifts, high energy intake and a greatly reduced fasting window when night shifts were incorporated within work schedules [24]. These findings suggest a rotating schedule may have a more profound impact on the eating habits and overall energy intakes of rotating shift workers due to perpetually changing work schedules. Currently, there are no large studies that examine dietary intakes according to fixed night versus rotating shift schedules. Fixed night shift work, defined as night work regularly performed to include the period between midnight and 5am [25] is reported to increase the risk of developing overweight/obesity by 29% compared to rotating shift work [26] and night shift and rotating shift schedules have been associated with higher risk of metabolic syndrome (MetS) and T2D [6, 27, 28]. While a number of factors including stress and deficits in sleep duration/quality on account of sleep–wake disruption imposed by shift work have been linked to higher prevalence of obesity and T2D in shift working populations [14, 29–31], unhealthy eating habits and mistimed eating during the night may also play a mediating role in adverse metabolic health outcomes [13, 32, 33]. Current national nutrition guidelines do not address the altered eating behaviours of shift workers and a recent review has identified a critical lack of effective weight management interventions for shift workers, due in part to oversight of relevant and commonly reported enablers and barriers to eating within shift working populations [34]. Emerging research on the feasibility of minimising night time eating amongst night shift working participants via time restricted eating [35] or implementing an overnight fast between 0100 to 0600 h [36] have shown these as promising strategies to improve weight management and cardiometabolic health. The benefits of overnight fasting have been recognised in the American Heart Foundation’s position statement on strategies to reduce caloric intake and potentially manage weight to mitigate CVD risk [37], however national dietary guidelines to date have no or limited consideration for food timing.

As both rotating and night shift schedules are associated with unintended metabolic health outcomes, it is important to explore the dietary profiles of workers undertaking these shift types in order to intervene with targeted dietary strategies. We hypothesize that dietary intakes of night shift workers will fail to meet national dietary recommendations and that those working a rotating schedule would have poorer dietary profiles than those working a fixed night schedule. The primary aim of the current study was to investigate habitual dietary intakes (daily energy, macronutrients, fibre, saturated fat, added sugar, sodium and alcohol), as compared to Australian dietary recommendations, of a large night shift working population (SWIFt trial [38]) characterised by overweight/obesity and working across numerous industries. Dietary intakes were analysed, controlling for age, gender, BMI, physical activity, years of shift work exposure, occupation and shift work schedule. As a secondary aim, dietary profiles were explored and compared according to rotating and fixed night shift schedules. Currently, national dietary guidelines are not well placed to incorporate emerging knowledge regarding the negative impact of meal timings on poorer health outcomes of shift workers. Findings from this study will contribute towards future direction on dietary based recommendations, aimed at improving shift worker health.

## Methods and materials

### Study design and population

This cross-sectional study utilises baseline data from a three-arm-parallel randomised controlled trial examining dietary weight loss in night shift workers (Shifting Weight using Intermittent Fasting in Shift Workers; SWIFt trial) [38]. Baseline data were collected between September 2019 and February 2021 at two Australian university facilities; Monash University in Melbourne, and the University of South Australia in Adelaide. Ethics approval for the SWIFt trial was granted by Monash Health Human Research Ethics Committee (RES 19–0000-462A), the University of South Australia (HREC ID: 202,379) and Ambulance Victoria Research Committee (R19-037) and registered with Australian New Zealand Clinical Trials Registry (ACTRN-12619001035112).

Trial participants were shift workers aged 25–65 years, undertaking at least two night shifts each week. To be eligible for the SWIFt trial, participants had to have worked night shifts for a minimum of six consecutive months. Due to the weight loss intervention study design, a BMI cut-off of > 26 kg/m^2^ was required for participants of Asian ethnicity, and > 28 kg/m^2^ applied to non-Asian shift workers. Exclusion applied to participants with a pre-existing medical condition such as CVD or T2D. Other factors known to impact on weight or participation in a weight loss trial were applied as exclusion criteria, as described in the protocol paper [38].

### Individual and lifestyle characteristics

Demographic variables were collected using an online screening questionnaire and included ethnicity, gender and date of birth. Height and body weight were measured as per the SWIFt trial protocol [38], and BMI calculated according to weight(kg)/height(m^2^). Participants completed the self-administered long form version of the International Physical Activity Questionnaire (IPAQ) [39, 40], a validated questionnaire assessing frequency and duration of physical activity (as MET minutes/week) performed over the past seven days. The IPAQ data were cleaned and processed using the 2005 guidelines for long forms [41], where reported activity deemed unfeasible were excluded by applying a cut off to physical activity exceeding 16 h per day spent in vigorous/moderate physical activity and walking when averaged across a week. Additionally to IPAQ, self-reported frequency, type and intensity of physical exercise performed on a weekly basis were collected. A researcher utilised physical activity data in conjunction with occupational information, to categorise Physical Activity Level (PAL) as 1.4, 1.6 and 1.8 for sedentary, light activity and moderate activity respectively based on the Food and Agriculture Organization and Black et al.[42, 43].

Occupation groups were obtained from a general shift work questionnaire previously developed for a randomised cross-over trial in night shift workers[44], which participants completed according to current shift work schedule, job title and shift work history. Shift schedules were clearly identified from a 14-day work diary, with fixed night shifts defined as working night shifts only, and rotating shifts defined by changing/different shift types inclusive of night shifts. Work industries represented by participant responses were categorised according to the Australian and New Zealand Standard Classification of Occupations [45].

### Dietary intake and misreporting of energy

Participants completed a 7-day food diary recorded either electronically via ‘Research Food Diary App’ (Xyris Software Pty Ltd, Australia), or in a handwritten proforma paper version. The food diary recording period included work and non-work days and occurred in the weeks prior to randomisation into a dietary weight loss study arm. Participants were instructed to follow their usual diet during the recording period and were given verbal and written instructions to complete the food diary using a booklet to identify and visualise portion sizes consumed. Data were entered into Foodworks Version 10 [46] for handwritten food diaries or imported for App based food diaries, using the Australia Diet and Recipes Analysis (AUSFOODS 2019) database. Foodworks contains an extensive database of food and beverage items, however newly developed food products not present required substitution with an equivalent product. Each food diary was visually checked and corrected with participants by a research dietitian at the baseline appointment of the trial, ensuring missing data (e.g. cooking style, condiments, brand names, serve sizes/quantities) were added. A dietary data collection protocol (see Supplementary Information S1) was used for data consistency. Data extracted from the Foodworks dietary analysis included average daily energy intake (kJ/day), macronutrients (g/day, percent total energy intake (%EI)), fibre (g/day), and nutrients with negative health associations (negative nutrients) namely alcohol (g/day,%EI), saturated fat (g/day,%EI), added sugar (g/day,%EI) and sodium (mg/day). Dietary data were compared against the Nutrient Reference Values for Australia and New Zealand (NRV) [47], in addition to the World Health Organisation recommendation for added sugar [48] (Table 1), to interpret dietary intakes against recommended daily intakes for Australian adults.

**Table 1:** Dietary recommendations for Australian adults [47, 48]

	Recommended daily intake
Protein	15–25% EI
Fat	20–35% EI
Saturated fat	< 10% EI
Carbohydrate	45–65% EI
Added sugar	< 10% EI
Alcohol	< 5% EI
Sodium	< 2000 mg
Fibre	
Females	25 g
Males	30 g

EI, energy intake

The revised Goldberg cut off [49] was applied to assess plausibility of reported energy intake (rEI) using a ratio with basal metabolic rate (BMR) and comparing the PAL for individuals (rEI:BMR and PAL), incorporating 95% confidence intervals (2 standard deviations) via the following equations:

$$S= \sqrt{\frac{{CV}^{2}rEI}{d}+{CV}^{2}BMR+{CV}^{2 }PAL}$$

*S* is the factor used for: within subject variation in reported energy intake (*rEI)*, number of days of dietary assessments completed (*d*), within subject variation in estimated BMR (*CVBMR*), and within subject variation in PAL (*CVPAL*). Based on Black’s recommendations[49], values used were CVrEI = 23%, CVBMR = 8.5%, CVPAL = 15%. After S was determined, the following equation was applied to dietary intake data:

$$cut \, offs \, for \, EIr:BMR \, and \, PAL \, value \times exp \left(\pm 2 SD\times \frac{S/100}{\sqrt{n}}\right)$$

PAL values for sedentary, light activity and moderate activity were applied as previously described. Misreporting was identified at the individual level, using n = 1.

### Statistical analyses

Analyses were conducted with the Statistical Package for Social Sciences (IBM SPSS Advanced Statistics, Version 28.0, Armonk, NY, IBM Corp.). Participant demographic and work characteristics (N = 250) were analysed using descriptive statistics, tested for normality and presented as mean (standard deviation), otherwise median (interquartile range) were reported. For dietary variables (n = 245), mean %EI of protein, fat, carbohydrates, saturated fat, added sugar and alcohol were compared to dietary recommendations and expressed as the proportion of participants consuming “below/within/above” recommended daily intakes. To check for underreporting, dietary intake data was assessed using the Goldberg cut off where both dietary data and PAL were provided (n = 223). Intakes of energy, macronutrients and negative nutrients were compared between plausible energy intake reporters (n = 132), and those designated as misreporters (n = 113) using unpaired t-tests. Removal of misreporters may create bias by excluding participants with unique characteristics and reduce statistical power [50, 51], therefore presenting separate energy intake data for the total sample containing misreporters (n = 245), and plausible reporters only (n = 132) was chosen to assist in interpretation of dietary data.

Prior to regression analysis, data were checked for assumptions of independence of observations, linearity, homoscedasticity, multicollinearity, presence of outliers and normality of continuous variables before proceeding. Hierarchical regression was chosen to control for the effects of independent variables on dietary intake and to account for possible causal effects of particular variables when predicting dietary intake and interpreting data. This regression method examined the effect of participant characteristics (age, gender, BMI, physical activity (MET mins/week), years of shift work exposure, occupation and shift schedule) on dietary intakes (total energy, macronutrients and negative nutrients). We used “exclude pairwise” in the regression analysis to maximise use of available data for participants with missing values, and after visually checking histograms for distribution, used Cook’s distance to check for outliers. Alcohol intake data (%EI) was excluded since many participants reported not consuming alcohol (n = 116, 47.3%), resulting in positively skewed data. Physical activity (MET minutes/week) data (n = 6 excluded as outliers) were transformed to squared root data due to large variation and non-normal distribution of the values, allowing a total of 15 outliers to be included in analysis (final sample n = 244). Additionally, the independent variable, occupation, was recoded as five dummy variables and each occupation category was compared to “professional” in the model due to having the largest sample size and greatest variation of all occupation groups. Hierarchical linear regression was conducted with available matching individual and lifestyle data (n = 237) for dependent dietary outcome variables (energy intake (kJ), macronutrients (%EI) and negative nutrients (%EI)), excluding alcohol. Four models were constructed with the following predictors/independent variables:

Model 1: age, gender.

Model 2: age, gender, BMI, physical activity.

Model 3: age, gender, BMI, physical activity, years of shift work exposure, occupation.

Model 4: age, gender, BMI, physical activity, years of shift work exposure, occupation, shift schedule.

The four models were repeated for plausible energy intakes (n = 127) to assess whether significant predictors for energy intake differed from the regression analysis containing misreporters.

**Fig. 1:**
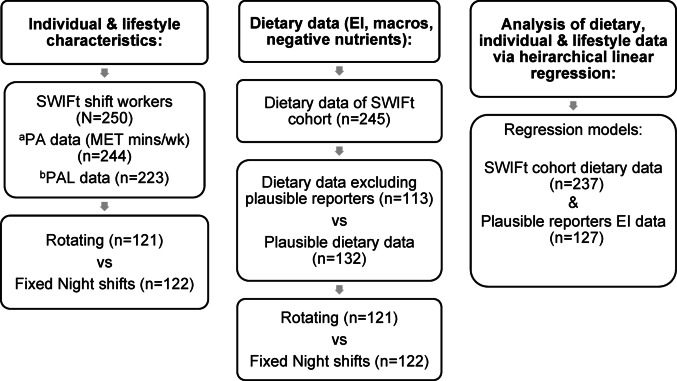
Flow diagram of samples and analyses used in the SWIFt trial. EI: energy intake; macros: macronutrients; ^a^PA: physical activity (MET minutes/wk, 6 outliers excluded); ^b^PAL: physical activity level (sedentary = 1.4, light = 1.6, moderate = 1.8) not reported by 27 participants

Differences in dietary intake data were compared between rotating and fixed night shift groups. Unpaired t-tests or Mann–Whitney tests were used for continuous variables (age, weight, BMI, shift work exposure, physical activity, total daily energy intake, macronutrients, negative nutrients, fibre and sodium) and chi-squared tests for comparisons of categorical variables (gender, BMI category, occupation groups, underreporting/plausible reporting, and percent below/within/above nutritional recommendations). A *p*-value of < 0.05 was taken as significant. Figure 1 displays a flow diagram of participants from the SWIFt trial included in the analyses reported.

## Results

In total, 1218 participants expressed interest in the SWIFt Trial, and 443 were eligible and invited to attend in-person screening interviews. Of these, 250 participants met the study criteria of the SWIFt trial and provided written informed consent for inclusion. The mean age of participants was 46.8 ± 9.8 years and 53% were female (Table 2). A high proportion of the cohort were classified with obesity (82.4%) and the median years of shift work exposure was 14.5 years (IQR:7.0–22.8).

**Table 2:** Characteristics of SWIFt trial participants

	Total (N = 250)
Age (y)	46.8 ± 9.8
Female, n (%)	133 (53)
Male, n (%)	117 (47)
Weight (kg)	101.0 ± 20.4
*Ethnicity, n (%)*	
European	168 (67.2)
Asian	21 (8.4)
Oceania	7 (2.8)
North African and Middle Eastern	3 (1.2)
People of the Americas	2 (0.8)
Other^a^	49 (19.6)
BMI (kg/m^2^)	34.8 ± 5.9
Overweight^b^ n (%)	44 (17.6)
Obese^a^ n (%)	206 (82.4)
*Shift schedule, n (%)*	
Fixed night	125 (50.0)
Rotating	123 (49.2)
Split	2 (0.8)
Shift work exposure (y)	14.5 [7.0–22.8]
*Occupation, n (%)*	
Managers	5 (2.0)
Professionals	86 (34.4)
Technicians/trades	27 (10.8)
Community/personal service	69 (27.6)
Clerical/administrative	22 (8.8)
Sales	2 (0.8)
Machinery operators/drivers	20 (8.0)
Labourers	19 (7.6)
Raw physical activity (MET mins/wk)^c^	5871 [2522–11423]

Values are means ± SD, median [IQR] or number (%)

^a^Other: respondents chose “other” and listed ethnicity as Australian (n = 35), African (n = 2), Multi-racial background (n = 2), Filipino (n = 2), Asian (n = 1), Caucasian (n = 1), Fijian (n = 1), Mauritian (n = 1), New Zealand (n = 1), South African (n = 1), South America (n = 1), or Not stated (n = 1)

^b^Asian BMI cut points (overweight = 23–27.5 kg/m^2^; obesity ≥ 27.5 kg/m^2^) and non-Asian BMI cut points (overweight = 25–29.9 kg/m^2^; obese ≥ 30 kg/m^2^)

^c^Physical activity: n = 244

### Dietary intakes and nutritional recommendations

The mean total daily energy intake of SWIFt cohort participants (n = 245) was 8716 ± 2765 kJ/day (Table 3). Average energy intakes from macronutrients were within NRV recommendations for protein (19.2 ± 3.9%), above requirements for total fat (35.8 ± 5.9%) and below the NRV for carbohydrate (41.4 ± 7.3%). Fibre intakes for females and males were both inadequate (19.1 ± 6.9 g/day and 22.6 ± 8.7 g/day respectively) and sodium exceeded recommendations (2742 ± 1091 mg/day). The majority of shift workers had acceptable intakes of added sugar and alcohol as percentages of daily energy intake (75.5% and 84.9% of participants respectively). More than two thirds of participants (69.0%) had carbohydrate intakes below recommended NRVs, and half (49.8%) exceeded requirements for total fat intake. Saturated fat intakes were above recommendations for most of the cohort (91.0%).

**Table 3:** Dietary intake comparisons with and without plausible reporters (PR)

	Total^a^	Plausible reporters^b^ (PR) (n = 132)	Total *without* PR^c^	*p*-value^bc^
	(n = 245)		(n = 113)	
Energy (kJ/day)	8716 ± 2765	10,206 ± 2421	6976 ± 2033	< **0.001**^ϕ^
Protein (g/day)	97.4 ± 33.0	109.3 ± 33.4	83.5 ± 26.5	< **0.001**^ϕ^
Protein (%) *ref* = *15–25%*	19.2 ± 3.9	18.1 ± 3.2	20.5 ± 4.3	< **0.001**^ϕ^
Below, n (%)	32 (13.1)	20 (15.2)	12 (10.6)	**0.012**‡
Within, n (%)	200 (81.6)	110 (83.3)	90 (79.6)	
Above, n (%)	13 (5.3)	2 (1.5)	11 (9.7)	
Total fat (g/day)	85.1 ± 32.7	101.1 ± 30.9	66.5 ± 23.7	< **0.001**^ϕ^
Total fat (%) *ref* = *20–35%*	35.8 ± 5.9	36.5 ± 5.8	35.1 ± 6.0	0.065
Below, n (%)	1 (0.4)	1 (0.8)	0 (0)	**0.035**‡
Within, n (%)	122 (49.8)	57 (43.2)	65 (57.5)	
Above, n (%)	122 (49.8)	74 (56.1)	48 (42.5)	
Saturated fat (g/day)	32.4 ± 13.6	38.5 ± 13.0	25.2 ± 10.3	< **0.001**^ϕ^
Saturated fat (%) *ref* < *10%*	13.6 ± 3.1	13.9 ± 3.0	13.2 ± 3.1	0.095
Within, n (%)	22 (9.0)	6 (4.5)	16 (14.2)	**0.009** ^**†**^
Above, n (%)	223 (91.0)	126 (95.5)	97 (85.8)	
Carbohydrate (g/day)	210.7 ± 71.7	246.2 ± 65.1	169.2 ± 55.0	** < 0.001** ^ϕ^
Carbohydrate (%) *ref* = *45–65%*	41.4 ± 7.3	41.4 ± 7.5	41.4 ± 7.1	0.987
Below, n (%)	169 (69.0)	95 (72.0)	74 (65.5)	0.332
Within, n (%)	76 (31.0)	37 (28.0)	39 (34.5)	
Above, n (%)	0 (0)	0 (0)	0 (0)	
Added sugar (g/day)	32.8 [19.2–52.1]	40.8 [24.3–62.3]	24.5 [16.2–38.0]	** < 0.001** ^χ^
Added sugar (%) *ref* < *10%*	6.6 [4.0–9.8]	6.7 [4.0–10.6]	6.1 [3.9–9.3]	0.274
Within, n (%)	185 (75.5)	95 (72.0)	90 (79.6)	0.182
Above, n (%)	60 (24.5)	37 (28.0)	23 (20.4)	
Alcohol (g/day)	0.0 [0.0–8.3]	1.7 [0.0–11.9]	0.0 [0.0–2.5]	** < 0.001** ^χ^
Alcohol (%) *ref* < *5%*	0.0 [0.0–2.7]	0.4 [0.0–3.5]	0.0 [0.0–1.3]	**0.005** ^χ^
Within, n (%)	208 (84.9)	108 (81.8)	100 (88.5)	0.157
Above, n (%)	37 (15.1)	24 (18.2)	13 (11.5)	
Fibre (g/day) *ref* = 25 g F	19.1 ± 6.9	22.1 ± 6.9	15.9 ± 5.5	** < 0.001** ^ϕ^
*ref* = 30 g M	22.6 ± 8.7	25.7 ± 8.9	18.2 ± 6.7	** < 0.001** ^ϕ^
Sodium (mg/day) *ref* < 2000 mg	2742 ± 1091	3089 ± 1079	2337 ± 961	< **0.001**^ϕ^

Values are means ± SD, median [IQR] or number (%). F; female, M; male, *ref*; recommended daily intakes according to NRV and WHO. Values in bold indicate a statistically significant result

^a^Total sample of SWIFt participants with dietary data (n = 245)

^b^Plausible reporters, with dietary and PAL data (n = 132)

^c^Total dietary data sample with plausible reporters removed (n = 113)

^ϕ^Levene’s Test for parametric continuous variables (*p* < 0.05)

^†^Pearson’s chi-square for categorical variables (*p* < 0.05)

^‡^Fisher’s Exact Test for categorical variables (*p* < 0.05)

^χ^Mann-Whitney U Test for non-parametric continuous variables (*p* < 0.05)

### Dietary intakes and misreporting energy intake

Of the 250 participants, 223 (89.2%) provided dietary and PAL data for Goldberg cut-offs to be applied. The 7-day food diary demonstrated underreported energy intake in 40.8% (n = 91) of individuals, and 132 (59.2%) were deemed plausible reporters. Female participants were more likely to underreport energy intake than males (56%, 44% respectively), and those who underreported had a higher BMI on average, compared to those with plausible reporting (35.7 ± 6.4 kg/m^2^ vs 33.6 ± 4.8 kg/m^2^). Energy intake (Table 3) was significantly higher amongst plausible reporters (10,206 ± 2421 kJ/day vs 6976 ± 2033 kJ/day, *p* < 0.001). As a percentage of energy intake, plausible reporters had lower protein intakes (18.1 ± 3.2% vs 20.5 ± 4.3%, *p* < 0.001) and higher alcohol intakes (0.4% IQR:0.0–3.5 vs 0.0% IQR:0.0–1.3, *p* = 0.005). Percentage energy intakes for total fat, carbohydrate and added sugar were similar. Mean sodium and fibre intakes were not within recommendations for either group but were significantly higher in plausible reporters.

### Effects of individual and lifestyle factors on dietary intakes of shift workers

#### Energy intakes (Model 4)

Females reported lower energy intakes than males in analyses including all participants (B =  − 2282 kJ; ꞵ =  −0.419; *p* < 0.001) (Table 4) and only plausible reporters ((B =  − 2557 kJ; ꞵ =  −0.538; *p* < 0.001) (Table 5), after controlling for all other individual and lifestyle factors. In plausible reporters (n = 127), increased BMI (B = 123 kJ; ꞵ = 0.235; *p* = 0.002) and a rotating shift schedule compared to fixed (B = 1006 kJ; ꞵ = 0.212; p = 0.011) were also identified as predictors of energy intake (Table 5). No other participant characteristics were significantly associated with energy intake.

**Table 4:** Hierarchical regression predicting energy intake (kJ) of all shift workers (n = 237)

Variable	Model 1		Model 2		Model 3		Model 4	
	B	ꞵ	B	ꞵ	B	ꞵ	B	ꞵ
Constant	11,304**		10,394**		9897**		9313**	
Age	− 30.3	− 0.108	− 27.3	− 0.097	− 18.9	− 0.067	− 13.2	− 0.047
Gender^a^	− 2245**	− 0.412**	− 2273**	− 0.417**	− 2371**	− 0.435**	− 2282**	− 0.419**
BMI			28.0	0.061	29.2	0.063	32.1	0.070
Physical activity^b^			− 2.32	− 0.032	− 2.67	− 0.037	− 2.62	− 0.037
Shift work exposure					− 1.15	− 0.005	− 3.25	− 0.013
Community/personal service^c^					732	0.121	500	0.083
Technicians/trades^c^					− 387	− 0.041	− 521	− 0.056
Managers/clerical/sales^c^					273	0.031	129	0.015
Machinery operators/ drivers^c^					− 496	− 0.051	− 576	− 0.059
Labourers^c^					96.7	0.009	9.39	0.001
Shift schedule^d^							614	0.113
*∆R* ^*2*^				0.005		0.022		0.011
*R* ^*2*^		0.184		0.189		0.210		0.221
*∆F*		26.3**		0.722		1.03		3.05
*F model*		26.3**		13.5**		6.02**		5.80**

Unstandardized (B) and standardized (ꞵ) regression coefficients are shown

Model 1: age and gender; Model 2: age, gender, BMI and physical activity; Model 3: age, gender, BMI, physical activity, years of shift work exposure and occupations; Model 4: age, gender, BMI, physical activity, years of shift work exposure, occupations and shift schedule. Values in bold indicate a statistically significant result

^a^0 = male, 1 = female

^b^Physical activity in MET minutes/week transformed into square root values

^c^ “professional” occupational group used as reference

^d^0 = fixed night shift, 1 = rotating shift

*p < 0.05; **p < 0.001

**Table 5:** Hierarchical regression predicting plausible energy intake (kJ) of shift workers (n = 127)

Variable	Model 1		Model 2		Model 3		Model 4	
	B	ꞵ	B	ꞵ	B	ꞵ	B	ꞵ
Constant Age Gender^a^	13292**		8966**		8643**		7958**	
	− 40.2*	− 0.159*	− 43.2	− 0.171*	− 42.7	− 0.169	− 35.0	− 0.139
	− 2490**	− 0.524**	− 2532**	− 0.533**	− 2495**	− 0.525**	− 2557**	− 0.538**
BMI			112*	0.215*	119*	0.229*	123*	0.235*
Physical activity^b^			9.6	0.146	7.9	0.121	7.8	0.118
Shift work exposure					2.9	0.013	4.5	0.020
Community/personal service^c^					647	0.123	72.3	0.014
Technicians/trades^c^					518	0.061	161	0.019
Managers/clerical/sales^c^					− 134	− 0.017	− 354	− 0.045
Machinery operators/ drivers^c^					− 485	− 0.057	− 953	− 0.113
Labourers^c^					− 788	− 0.089	− 1138	− 0.129
Shift schedule^d^							1006*	0.212*
*∆R* ^*2*^				0.055		0.035		0.033
*R* ^*2*^		0.306		0.361		0.396		0.429
*∆F*		27.3**		5.29*		1.11		6.69*
*F model*		27.3**		17.2**		7.59**		7.85**

Unstandardized (B) and standardized (ꞵ) regression coefficients are shown

Model 1: age and gender; Model 2: age, gender, BMI and physical activity; Model 3: age, gender, BMI, physical activity, years of shift work exposure and occupations; Model 4: age, gender, BMI, physical activity, years of shift work exposure, occupations and shift schedule. Values in bold indicate a statistically significant result

^a^ 0 = male, 1 = female

^b^ Physical activity in MET minutes/week transformed into square root values

^c^ “professional” occupational group used as reference

^d^ 0 = fixed night shift, 1 = rotating shift

*p < 0.05; **p < 0.001

#### Macronutrient intakes (Model 4)

Although the overall, fully adjusted models did not significantly predict macronutrient intakes, increased physical activity was related to higher protein intake (B = 0.18%, ꞵ = 0.176, *p* = 0.009) (Supplementary Information S2), and lower fat intake (B =  − 0.025%, ꞵ =  − 0.162, *p* = 0.016) (Supplementary Information S3) and being a labourer was related to lower carbohydrate intake compared to professional occupations (B =  − 4.48%, ꞵ =  − 0.165, *p* = 0.023) after controlling for all other individual and work-related predictors (Supplementary Information S4).

#### Negative nutrient intakes (Model 4)

Increases in physical activity were related to lower saturated fatty acid (SFA) intake, (B =  − 0.013%, ꞵ =  − 0.165; *p* = 0.014) (Supplementary Information S5) but not added sugar intake after controlling for individual and work-related factors (Supplementary Information S6). Increasing age was associated with decreased added sugar intake however this association was not significant when shift work exposure was added to the model.

### Characteristics and diets of shift workers (rotating versus fixed night shift schedules)

Of the 250 study participants, 50% (n = 125) worked fixed nights (FN), 49.2% (n = 123) worked rotating shifts (R) and 0.8% (n = 2) in split shifts (Table 2). Rotating shift workers were younger (45.2 ± 10.1 years vs 48.5 ± 9.2 years), more likely to be male (58.7% vs 35.2%) and a greater proportion worked in community/personal service (41.3%) while a higher proportion of fixed night workers undertook professional roles (50.8%) (Table 6).

**Table 6:** Characteristics and dietary intakes of rotating vs fixed night shift workers

	Rotating (R)	Fixed night (FN)	*p*-value
	(n = 121)	(n = 122)	(R vs. FN)
*Individual and lifestyle characteristics*			
Age (y)	45.2 ± 10.1	48.5 ± 9.2	**0.009**
Female, n (%)	50 (41.3)	79 (64.8)	** < 0.001** ^†^
Male, n (%)	71 (58.7)	43 (35.2)	
Weight (kg)	102.6 ± 18.1	98.3 ± 21.7	0.093
BMI (kg/m^2^)	34.3 ± 5.2	35.0 ± 6.5	0.354
Overweight^a^ n (%)	20 (16.5)	23 (18.9)	0.759
Obese^a^ n (%)	101 (83.5)	99 (81.1)	
Shift work exposure (y)	13.0 [7.0–21.5]	15.1 [5.8–25.3]	0.43
Occupation, n (%)			
Managers	3 (2.5)	1 (0.8)	** < 0.001** ^‡^
Professionals	23 (19.0)	62 (50.8)	
Technicians/trades	14 (11.5)	11 (9.0)	
Community/personal service	50 (41.3)	18 (14.8)	
Clerical/administrative	11 (9.1)	10 (8.2)	
Sales	0 (0)	1 (0.8)	
Machinery operators/drivers	10 (8.3)	10 (8.2)	
Labourers	10 (8.3)	9 (7.4)	
Physical activity (MET mins/wk)^b^	6255 [2988–10720]	5853 [2264–12556]	0.777
*Dietary intake*			
Under reporting, n (%)^c^	43 (39.4)	48 (42.9)	
Plausible reporting, n (%)^c^	66 (60.6)	64 (57.1)	0.607
Plausible energy (kJ/day)^c^	10,968 ± 2411	9307 ± 2070	** < 0.001** ^ϕ^
Energy (kJ/day)	9329 ± 2915	8025 ± 2383	** < 0.001** ^ϕ^
Protein (g/day)	103.9 ± 33.6	89.9 ± 30.1	** < 0.001** ^ϕ^
Protein (%) *ref* = *15–25%*	19.3 ± 4.1	19.2 ± 3.8	0.94
Below, n (%)	18 (14.9)	14 (11.5)	0.686
Within, n (%)	96 (79.3)	102 (83.6)	
Above, n (%)	7 (5.8)	6 (4.9)	
Total fat (g/day)	91.8 ± 35.3	77.6 ± 27.6	** < 0.001** ^ϕ^
Total fat (%) *ref* = *20–35%*	36.0 ± 6.2	35.6 ± 5.8	0.647
Below, n (%)	0 (0)	1 (0.8)	0.848
Within, n (%)	62 (51.2)	60 (49.2)	
Above, n (%)	59 (48.8)	61 (50.0)	
Saturated fat (g/day)	34.9 ± 14.3	29.4 ± 11.6	**0.001** ^ϕ^
Saturated fat (%) *ref* < *10%*	13.7 ± 3.0	13.5 ± 3.2	0.611
Within, n (%)	12 (9.9)	10 (8.2)	0.64
Above, n (%)	109 (90.1)	112 (91.8)	
Carbohydrate (g/day)	222.1 ± 75.3	198.0 ± 65.2	**0.008** ^ϕ^
Carbohydrate (%) *ref* = *45–65%*	40.8 ± 7.4	42.0 ± 7.1	0.186
Below, n (%)	90 (74.4)	77 (63.1)	0.079
Within, n (%)	31 (25.6)	45 (36.9)	
Above, n (%)	0 (0)	0 (0)	
Added sugar (g/day)	34.4 [21.0–53.4]	29.3 [16.7–48.5]	0.087
Added sugar (%) *ref* < *10%*	6.8 [4.2–9.7]	6.2 [3.9–10.1]	0.663
Within, n (%)	93 (76.9)	91 (74.6)	0.793
Above, n (%)	28 (23.1)	31 (25.4)	
Alcohol (g/day)	1.9 [0.0–12.3]	0.0 [0.0–3.9]	**0.005** ^χ^
Alcohol (%) *ref* < *5%*	0.5 [0.0–3.3]	0.0 [0.0–1.8]	**0.010** ^χ^
Within, n (%)	96 (79.3)	111 (91.0)	**0.018** ^†^
Above, n (%)	25 (20.7)	11 (9.0)	
Fibre (g/day) *ref* = 25 g F	20.0 ± 6.9	18.5 ± 6.9	0.221
*ref* = 30 g M	23.8 ± 8.8	20.6 ± 8.3	0.06
Sodium (mg/day) *ref* < 2000 mg	2923 ± 1077	2519 ± 1023	**0.003** ^ϕ^

Values are means ± SD, median [IQR] or number (%). F; female, M; male, *ref*; recommended daily intakes according to NRV and WHO. Values in bold indicate a statistically significant result

^a^Asian BMI cut points (overweight = 23–27.5 kg/m^2^; obesity ≥ 27.5 kg/m^2^) and non-Asian BMI cut points (overweight = 25–29.9 kg/m^2^; obese ≥ 30 kg/m^2^)

^b^Physical activity: n = 117 for Rotating (R), n = 120 for Fixed Night (FN)

^c^Goldberg cut-offs were applied to 7 day dietary data using available PAL data

^ϕ^Levene’s Test for parametric continuous variables (*p* < 0.05)

^†^Pearson’s chi-square for categorical variables (*p* < 0.05)

^‡^Fisher’s Exact Test for categorical variables (*p* < 0.05)

^χ^Mann-Whitney U Test for non-parametric continuous variables (*p* < 0.05)

**Fig. 2:**
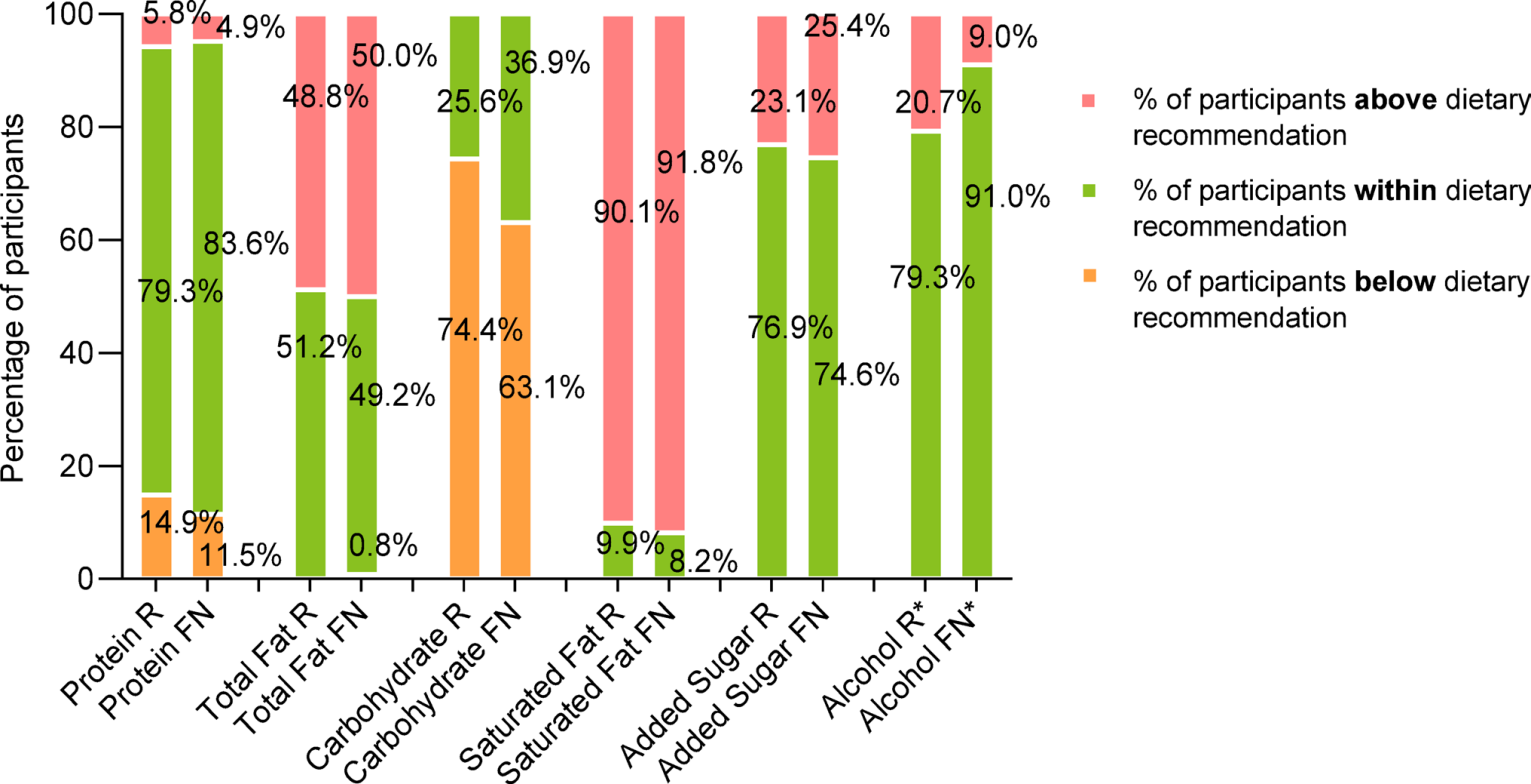
Percentages of rotating (R) and fixed night (FN) shift workers above, within or below each dietary recommendation for nutrient intakes. Note rotating (n = 121), fixed night (n = 122). *p < 0.05

Rotating shift workers reported significantly higher energy intakes compared to fixed night workers (R: 9329 ± 2915 kJ/day vs FN: 8025 ± 2383 kJ/day, *p* < 0.001) (Table 6). Mean values for macronutrient intakes were significantly higher amongst rotating shift workers but did not differ between groups when compared as percentage of total energy. Macronutrient intakes (%EI) for shift workers within each work schedule group (Fig. 2) showed similar trends and negative nutrient consumption for both groups were high for saturated fat intake, acceptable for added sugar and acceptable for alcohol intake. However, the rotating group had significantly higher alcohol intake (0.5% IQR:0.0–3.3 vs 0.0% IQR:0.0–1.8, *p* = 0.010) with a greater proportion exceeding recommendations for alcohol (R: 20.7% vs FN: 9.0%, *p* = 0.018). Sodium intake was in excess for both groups, and significantly higher amongst rotating shift schedules (2923 ± 1077 mg/day vs 2519 ± 1023 mg/day, *p* = 0.003).

## Discussion

This study investigated the habitual dietary intakes of night shift workers enrolled into the SWIFt trial, compared with Australian dietary recommendations. The study further explored differences in dietary profiles according to shift work schedules (rotating vs fixed night). As hypothesised, shift workers reported dietary intakes that did not meet national dietary recommendations for nutrients associated with chronic disease risk. Night shift workers had, on average, dietary profiles that had acceptable protein, added sugar and alcohol intakes but intakes of fat, saturated fat and sodium were higher than recommended. Furthermore, intakes of carbohydrate and fibre were lower than recommended. Further to our hypothesis, rotating shift workers reported higher average energy intakes, but also increased sodium and alcohol intake compared to fixed night shift workers.

Our cohort reported an average energy intake of 8716 kJ/day, although our data did exhibit high levels of underreporting. While controlling for individual and lifestyle factors, our study indicated male gender influenced higher energy intake across the entire cohort and within shift schedules. While male gender is a strong contributor to higher energy intake, it is important to note that in our study and in the literature, females are more prone to underreporting energy [52, 53].

When only plausible energy reporters were analysed for energy intake (n = 127), an increased BMI was a significant predictor of higher energy intake. In the literature, the magnitude of underreporting total energy intake is known to increase as body mass increases [50] and this was also evidenced in our study. An Australian study identified the proportion of under-reporting of energy intake in national nutrition surveys to increase according to increasing BMI categories, ranging from 20% for individuals within underweight and normal BMI categories to 65% for individuals within the obese category [54].

The relationship between BMI and energy intake is likely complex for shift workers. The impact of circadian disruption and mistimed energy intake at night, and its effects on decreased post-prandial insulin sensitivity and higher glucose response during night-time hours [55], may increase BMI in the absence of increased energy intake. Furthermore, the prioritisation of energy to the latter half of the biological day, as is common in night shift work, may exacerbate unintended weight gain and promote metabolic disorders.

In our study, shift schedule was an additional predictor of energy intake in plausible reporters. A recent systematic review and meta-analysis of 18 observational studies indicated rotating shift workers consume higher 24-h energy intake of 264 kJ/day compared to day workers [23], and reported intra-person energy intakes of rotating shift workers were similar on rotating day/morning shifts and rotating night shifts [23]. Additionally, a separate study conducted with 30 shift workers from the mining industry indicated 24-h energy intakes do not differ across a 10-day rotating shift cycle, but that each day demonstrated overconsumption of estimated energy requirements [24]. These combined findings suggest energy is consistently high throughout rotating shift schedules and may be characteristic of constantly changing day-to-night shifts.

Underlying physiological mechanisms behind energy intake and food preferences of shift workers are not yet well understood, but may be influenced by sleep restriction and chronic circadian disruption. Controlled in-laboratory research suggests acute sleep restriction of 1–2 nights increases hunger and appetite [56–58] whilst a longer sleep restriction duration of 5 days results in decreased levels of hunger and appetite but with increased food consumption when food access is provided ad libitum [59, 60]. A recent tightly controlled single blind in-laboratory study of 17 healthy participants randomised to control (n = 8) or chronic sleep restriction (n = 9) over 32 days reported an association between change in ghrelin/leptin ratio and decreased subjective hunger, appetite and food preference with chronic circadian disruption, suggesting circadian disruption rather than sleep deprivation may drive changes in eating behaviour [61]. However the study did not include rotating or night shift workers and provided a tightly controlled food environment and menu, so that findings may not directly apply to shift working populations with chronic circadian disruption.

Shift workers, regardless of shift type, were observed to have acceptable intakes of protein (19.2% EI). Our data is supported by other studies on shift workers whereby adequate protein intakes have been observed; an observational study combining fixed night (n = 27), 8-h rotating (n = 29) and 12-h rotating (n = 29) shift workers reported 19.2–20.7% for protein intakes [62] and a study on 22 night shift workers from a randomized cross-over trial reported baseline protein intake of 19.7% [10].

The average fat and saturated fat intakes observed in our cohort exceed those of the NRVs (35.8% EI for total fat, and 13.6% EI for saturated fat) and are similar to other studies where fat intake has been reported [10, 62, 63]. Whilst we did not observe an influence of gender or shift schedule, Heath et al. [62] demonstrated fixed night shift workers had higher total fat intake compared to rotating workers and Seychell and colleagues [63] reported females on rotating and fixed night shifts had higher fat intakes than their male counterparts. In our study lower levels of physical activity were predictive of higher fat intakes after controlling for individual and work-related factors. Of those who recorded physical activity levels, almost half (45.7%) reported sedentary levels of PAL, which has been linked to higher total fat and saturated fat intake in adult populations [64, 65]. Studies on shift working healthcare personnel report less physical activity on night shifts [66] or similar physical activity to their non-shiftworking counterparts [67] but significantly higher percentage of energy intake from fat [66, 67]. As excessive saturated fat intake presents additional cardiovascular risk alongside elevated postprandial lipid, glucose and circulating insulin levels associated with eating at night [17–19], this would suggest that restricting fat intake, particularly of saturated fats at night-time could be beneficial in reducing negative effects to metabolic health.

The low carbohydrate (41.4% EI), inadequate fibre (19.1 g/day and 22.6 g/day in women and men respectively) and higher fat diet observed in our cohort has implications for poorer shift worker health, particularly if derived from animal based sources as such diets promote oxidative stress and inflammatory pathways, and are associated with higher mortality rates [68]. Previous studies exploring carbohydrate intakes are variable with two studies in nurses finding higher carbohydrate intakes in shift workers compared to non-shift workers, although overall, intakes were low [69, 70]. A cross-sectional study of Australian shift workers from several industries suggests workers might switch between fat and carbohydrate to compensate for fatigue levels and sleep duration [62]. Our regression analysis identified lower reported carbohydrate intakes in labourers compared to those in professional roles, which may suggest a preference for fat intake amongst labourers that is associated with higher physical demands and work-related fatigue [62].

The relatively low (7.7%) percentage of energy intake from added sugar amongst the cohort was unexpected, considering previous studies on night shift workers have observed frequent snacking of discretionary foods high in sugar, fat and salt [9, 32, 71]. Furthermore, there were no significant predictors of added sugar intake in the SWIFt cohort. Two large cohort studies have reported high intakes of sugar sweetened beverages and/or confectionary in Japanese female nurses working rotating shift schedules compared to nurses working day schedules [7, 8]. However there has been a change towards “zero sugar” drinks and increased use of sugar alternatives in Australia [72], a trend also observed in the SWIFt cohort, which could explain lower added sugar intakes. Sodium intakes of shift workers were higher than recommended (2742 mg) and rotating shift workers were even less adherent than fixed night workers to the < 2000 mg/day guideline (2923 mg vs 2519 mg) [47]. While it is likely that higher sodium intake amongst rotating shift workers is directly related to the observed higher average energy consumption within the group, our findings suggest that dietary advice should focus on reducing sodium intake and the use of low salt alternatives.

Studies show that alcohol is used by shift workers to cope with issues such as stress, work-related fatigue and sleep disorders [7, 8, 73], yet we did not see high levels of alcohol intake in our cohort with 64.5% of participants reporting alcohol intakes of < 1% energy intake. However, in our cohort nearly a third (32.8%, 71 female, 11 male) of participants were nurses, most of whom worked fixed night shifts, which may have influenced lower alcohol intakes amongst this group compared to rotating workers, in line with the profession’s requirement to avoid alcohol intoxication for safe practice [74]. Furthermore, emergency workers such as paramedics and firefighters within the cohort have similar alcohol-free requirements in order to safely perform job tasks.

Overall, the findings of our study suggest that in our cohort of shift workers, a high proportion failed to meet national nutritional recommendations for key nutrients. As such, this dietary profile could contribute to the poorer metabolic health outcomes experienced by shift working populations. Longer years of exposure to shift work is associated with greater increases in body mass index compared to the normal trajectory of those undertaking day work, with each year contributing an increase of 0.12 kg/m^2^ (p < 0.05) [75, 76] and an overall higher BMI by 2.28 kg/m^2^ amongst night shift workers compared to day workers [77]. In particular, those in rotating shift work roles present with a diet higher in total energy intake, which may in the longer-term be a contributor to weight gain, obesity and worsening risk for T2D.

Our findings would suggest that an improved dietary profile for shift workers could be beneficial in reducing the negative effects to metabolic health particularly in terms of reducing total fat, saturated fat and sodium intake, and increasing dietary fibre to within NRV recommendations as a starting point. To achieve a more favourable dietary profile, practical recommendations for shift workers should emphasize greater intakes of fruit, vegetables, wholegrains, nuts, seeds and reduced fat dairy products with decreased intakes of fatty meats, processed and ultra-processed foods high in fat, saturated fat and sodium. Furthermore, restricting intake of saturated fatty acids and minimising energy intake during night shift hours by redistributing a greater proportion of food intake to before the biological night or before the start of the night shift is recommended. As the metabolic health of shift workers is exacerbated by the mistimed eating of suboptimal foods at night[15, 78], nutrition recommendations need to address altered eating habits while workplaces could improve access to better food options and facilities.

Our study displayed some unique strengths. The study population represented a comparative number of male/female shift workers in addition to a variety of work industries and occupations, providing valuable context and generalisability of dietary results for shift workers with overweight/obesity. Dietary data were assessed for underreporting to obtain a more accurate representation of shift workers’ diets, a step that is often overlooked in studies on shift workers, and the dietary intake regression analysis controlled for individual and lifestyle related factors. However, in this analysis, consideration of whether occupation contributed meaningfully to variation in dietary profiles was limited by the unequal size of the occupation categories. Further limitations to our study include the self-reported nature of dietary data collection, likely influenced by an observer effect whereby participants omit less healthy items or underreport portion sizes of foods and drinks consumed [52]. A limitation of Foodworks is the requirement to substitute reported foods or beverages with an equivalent item from the AUSFOODS 2019 database when specific brands and product names were not listed in the software, potentially limiting the accuracy of included foods. Furthermore, participants had enrolled in a weight loss trial, which may have influenced recall bias for physical activity (IPAQ and PAL) and dietary reporting. Even so, the levels of underreporting energy intake in our study (40.8%) were comparable to the 41% of underreporting observed in Australian non-shift working populations, using the same revised Goldberg cut-off approach [79].

## Conclusion

Our study has provided important insight into dietary profiles of shift workers. Shift worker diets were high in fat, saturated fat and sodium, while low in carbohydrate and fibre, regardless of shift schedule. In addition, the study identified an influence of rotating shift schedules on higher energy intakes, even after controlling for gender and BMI. As such, these findings continue to build on previous data highlighting inadequacies in the diet of shift workers and their increased risk of developing diet-related chronic disease, and identify opportunities to intervene. This study provides important recommendations to improve the dietary profiles of shift workers, and advice for the timing of energy consumption during night shifts. Future research on dietary intakes of shift workers should consider the combined impacts of altered meal timing, stress and altered sleep on the metabolic health of workers in order to identify further practical recommendations. Appropriate solutions for shift working populations are warranted to mitigate metabolic health risks, such as targeted dietary strategies and improvements to food access for healthier food choices within shiftworking environments.

## Supplementary Information

Below is the link to the electronic supplementary material.

Supplementary Material 1

## Abbreviations

BMI: Body mass index
CVD: Cardiovascular disease
EI: Energy intake
IPAQ: International physical activity questionnaire
MetS: Metabolic syndrome
PAL: Physical activity level
T2D: Type 2 diabetes
