# Supplementary material for: The swift study: dietary profiles of night shift workers characterised by overweight and obesity
Source: Eur J Nutr. 2026 Apr 10;65(3):115. doi: 10.1007/s00394-026-03917-9 (PMC13068731; doi:10.1007/s00394-026-03917-9)
Supplement: Supplementary file 1 — Supplementary Material 1 [file 394_2026_3917_MOESM1_ESM.docx]

**S1 DIETARY DATA PROTOCOL**

**SWIFt trial**

**PROCEDURE: Data Entry Checking - Foodworks**

**Written by:** Michelle Rogers (nee Headland)

**Date:** 09 February 2022

**Applicable to:** Monash University, Be Active Sleep and Eat (BASE) Facility,

Department of Nutrition, Dietetics and Food, Notting Hill, Victoria

University of South Australia, Clinical Trials Facility

City East Campus, South Australia

**AIM:** Provide instructions on how to record 7-day food diaries in Foodworks

**EQUIPMENT:** PC, Foodworks version 10

**RESPONSBILE:** All researchers involved in the SWIFt trial

**Introduction**

Foodworks is a trusted nutrition analysis software for dietitians, nutritionists, nutrition educators, food technologists and nutrition researchers, developed by Xyris Australia. Foodworks creates reports of the following nutrition information:

- Analysis of participants food records and 24-hour recalls
- Develops recipes and meal plans
- Assesses participants’ progress towards goals
- Uses the latest, most comprehensive food data for Australia and New Zealand

**Data Entry Checking – Participant 7 Day Food Diaries**

**Participant Food Diaries, either App or Paper version, should be entered into Foodworks by one study personnel, and checked by an alternative study personnel.**

1. Once the participant’s food diary has either been entered manually, or exported from the App into FoodWorks, all nutrient data needs to be GREY, with any food or quantities to be updated to remove any RED nutrient values.
2. Once entry has been completed, all entries are then checked by a second study personnel, ensuring the following:

- Food names/types selected reflect information provided by the participant^
- Quantities inputted reflect information provided by the participant^

^This information may also include any clarifications that were made by study personnel during data collection visits

When specific information regarding food or brands was not available in a participant’s diary the following selections were chosen (Table S1). Please note that these should only be used, if clarification cannot be sought from the participant directly, or if brand/specific details are not available on another occasions in the food diary.

Table S1. Generic Foods

| Generic Foods | Foodworks entry to be used | Suggested quantity if nil provided |
| --- | --- | --- |
| Toast | Bread,other,commercial,toasted | Regular slice |
| Bread | Bread,other,commercial,fresh  If white - Bread,white,commercial,unfortified,fresh | Regular slice |
| Hamburger Bun | Bread rolls, other, fresh | 1 Hamburger bun |
| Crumpet | Crumpet,regular,toasted | Round crumpet |
| Rice cake | Rice cake, from rice, plain | Thin cake |
| Wrap | Wrap, plain, white, fresh | Regular tortilla |
| Cracker | Cracker, other, other | Regular round cracker |
| Oats | Rolled oats,cooked,plain,unfortified,other |  |
| Porridge | Porridge,plain,unfortified,other |  |
| Corn flakes | Breakfast cereal, corn based, flakes, other |  |
| Pasta | Pasta,other,commercial,cooked |  |
| Rice | Rice,white,cooked,boiled |  |
| Cheese | Cheese,cheddar,other,regular fat | Sandwich slice |
| Cheese, fetta | Cheese, fetta, regular fat | Cracker-size slice |
| Milk | Milk,cow,ready to drink,other |  |
| Light Milk | Milk,cow,ready to drink,reduced fat,other |  |
| Chocolate milk | Flavoured milk, chocolate, other |  |
| Yoghurt | Yoghurt, commercial, natural, other |  |
| Coffee | Black- Coffee,instant coffee made up,black,caffeinated  White - Coffee,instant coffee made up,white,caffeinated,other  None specified – look at previous days OR Coffee,instant coffee made up,black,caffeinated | 1 cup |
| Black Tea | Tea,black,regular,plain,without milk | 1 cup |
| Sugar | Sugar,raw,regular |  |
| Red wine | Wine,red,regular | 150ml per glass |
| White wine | Wine,wine,regular | 150ml per glass |
| Wine | Wine,other | 150ml per glass |
| Cordial eg. Lemon | *Enter amount of water + 1 tbsp of cordial type* |  |
| “No sugar” softdrink | *If no option for zero/low/reduced sugar, chose “diet” version* |  |
| “No sugar” sports drinks | *If no option, choose no added sugar cordial “Cottee’s No added sugar lemon crush cordial” for similar kJ* |  |
| Cut of meat | *cut of meat*,semi trimmed,fried,other/added fat - if no cooking method or oil specified |  |
| Bacon | Bacon,breakfast rasher,fried,added fat | Rasher |
| Ham | Ham,fresh,leg,lean |  |
| Meatballs | Meatballs, beef, homemade, regular, fried, added fat | Regular meatball |
| Flake | Fish, flake, deep fried, coated | Medium fillet |
| Sausage | Sausage, other, fried | 1 long & thin sausage |
| Egg | Eggs,chicken,whole,fried,other,in oil | Medium OR regular |
| ‘Lean Cuisine’ | Check other days of food diary and choose same option  OR default: Satay chicken noodles 280g |  |
| “x brand” frozen meal | Look up brand and choose closest “Lean Cuisine” or “Jenny Craig” brand similar meal alternative with closest kJ, protein, fat, carbohydrate content. If no brand or type specified, or unable to find appropriate meal, use default; Lean Cuisine Satay Chicken Noodles 280g |  |
| Apple | If no colour provided - Apple,fresh,other,other,unpeeled  Red - Apple,fresh,other,red skin,unpeeled  Green - Apple,fresh,other,green skin,unpeeled | Medium |
| Banana | Banana,fresh,cavendish | Medium |
| Orange | Orange, other |  |
| Potato | Potato,plain,other,other,peeled, *then enter appropriate cooking method (microwave if nil)* | Medium |
| Carrot | Carrot,regular,fresh,peeled,microwaved,no added fat |  |
| Salad | Salad,garden,no additions |  |
| Asian Salad | Salad,asian style,without dressing |  |
| Cucumber | Cucumber, other, unpeeled, fresh |  |
| Lettuce | Lettuce,iceberg | 2-3 medium leaf |
| Tomato | Tomato, other, raw |  |
| Jam | Jam,other,regular | 1 regular spread |
| Vegemite | Spread, yeast, vegemite, regular |  |
| Peanut butter | Peanut butter, other |  |
| Butter | Butter,other |  |
| Margarine | Margarine,other,regular fat,other | Drizzle |
| Mayonnaise | Mayonnaise,commercial,other |  |
| Olives | Olives, black |  |
| Oil | Oil,other  If olive: Oil,olive,regular | Drizzle |
| Cream | Cream, other | Dollop |
| Ice cream |  | Medium scoop |
| Potato chips | Crisps,potato,regular,plain,salted |  |
| Potato cake | Potato scallop |  |
| Fries | Fries takeaway~restaurant~café, added salt | Regular takeaway |
| Meat pie | Pie, savoury, meat, topped with pastry, commercial, individual size |  |
| Brownie | Brownie, without nuts, other | Regular slice |
| Biscuit | Biscuit, sweet, commercial, plain |  |
| Mixed nuts salted | Mixed nuts, without dried fruit or seeds, +almond+cashew+peanut, salted |  |
| Pumpkin soup | Soup, pumpkin, homemade, prepared with stock | Bowl |
| Mashed potatoes | Potato, mashed, homemade, from fresh potato, other, peeled, with milk & butter |  |
| Roasted vegetables | Chose vegetable, other, unpeeled, fresh, roasted, other or added fat |  |
| SWIFt nut pack | If type of nut pack not specified or available in Foodworks, use default “SWIFt Lucky Oven Roasted almonds & cranberries” |  |
| SWIFt trial foods | For SWIFt trial food items, check if available in SWIFt trial food items list first. If not available in this list, choose most appropriate item as per instructions above or choose closest Foodworks item for energy content. |  |

^*^Select “other” when details for the component of that food are not specified

^*^Select “regular fat” when details of the type of fat content have not been specified

^**^Please note: when entering food data, if there is something that is unclear on any given day, please refer to the other days in participant food diaries to see if they have had the product option before and use those same details i.e. ‘Coffee’ on day 3 vs. Day 1 where they state exactly how they have their coffee; 2 Tsp Instant Coffee, 30ml Skim Milk, 125ml Skim Milk^**^

1. Once data entry has been checked by a second study personnel, data is saved and exported.
2. Once exported the following values are checked as part of a ‘common sense’ pass i.e. are these values likely:

- **Energy**
- **Fibre**
- **Caffeine**

1. If there are any values that are thought to be too high or too low, the individual’s FoodWorks data is then checked against the Raw Data file to check for any entry errors. Any errors are updated prior to the information being exported again.

Table S2 Hierarchical regression predicting protein intake (%EI) in shift workers (n= 237)

|  | **Model 1** | | **Model 2** | | **Model 3** | | **Model 4** | |
| --- | --- | --- | --- | --- | --- | --- | --- | --- |
| **Variable** | **B** | **ꞵ** | **B** | **ꞵ** | **B** | **ꞵ** | **B** | **ꞵ** |
| Constant  Age  Gender^a^ | 18.1^**^  .037  **-1.12^*^** | .092  **-.144^*^** | 15.0^**^  .030  **-1.03^*^** | .075  **-.133^*^** | 14.8^**^  .012  -.738 | .031  -.095 | 14.8^**^  .011  -.759 | .028  -.098 |
| BMI  Physical activity^b^ |  |  | .052  **.019^*^** | .080  **.189^*^** | .061  **.018^*^** | .093  **.176^*^** | .061  **.018^*^** | .092  **.176^*^** |
| Shift work exposure  Community/personal service^c^  Technicians/trades^c^  Managers/clerical/sales^c^  Machinery operators/ drivers^c^  Labourers^c^ |  |  |  |  | .027  .021  .730  -.283  1.15  .392 | .073  .002  .055  -.023  .083  .027 | .027  .077  .762  -.248  1.17  .413 | .074  .009  .057  -.020  .084  .028 |
| Shift schedule^d^ |  |  |  |  |  |  | -.148 | -.019 |
| *∆R^2^* |  |  |  | .038 |  | .010 |  | .000 |
| *R^2^* |  | .029 |  | .067 |  | .077 |  | .077 |
| *∆F* |  | 3.46^*^ |  | 4.72^*^ |  | .409 |  | .073 |
| *F model* |  | 3.46^*^ |  | 4.15^*^ |  | 1.89^*^ |  | 1.71 |

Unstandardized (B) and standardized (ꞵ) regression coefficients are shown.

Model 1: age and gender; Model 2: age, gender, BMI and physical activity; Model 3: age, gender, BMI, physical activity, years of shift work exposure and occupations; Model 4: age, gender, BMI, physical activity, years of shift work exposure, occupations and shift schedule. Values in **bold** indicate a statistically significant result.

^a^ 0 = male, 1 = female

^b^ Physical activity in MET minutes/week transformed into square root values

^c^“professional” occupational group used as reference

^d^ 0 = fixed night shift, 1 = rotating shift

^*^p < .05; ^**^p <.001

Table S3 Hierarchical regression predicting total fat intakes (%EI) in shift workers (n= 237)

|  | **Model 1** | | **Model 2** | | **Model 3** | | **Model 4** | |
| --- | --- | --- | --- | --- | --- | --- | --- | --- |
| **Variable** | **B** | **ꞵ** | **B** | **ꞵ** | **B** | **ꞵ** | **B** | **ꞵ** |
| Constant  Age  Gender^a^ | 34.5^**^  .023  .419 | .038  .035 | 31.6^**^  .046  .189 | .075  .016 | 31.8^**^  .057  -.163 | .094  -.014 | 31.3^**^  .063  -.079 | .103  -.007 |
| BMI  Physical activity^b^ |  |  | .121  **-.028^*^** | .121  **-.181^*^** | .097  **-.025^*^** | .096  **-.163^*^** | .099  **-.025^*^** | .099  **-.162^*^** |
| Shift work exposure  Community/personal service^c^  Technicians/trades^c^  Managers/clerical/sales^c^  Machinery operators/ drivers^c^  Labourers^c^ |  |  |  |  | -.002  .021  -1.95  1.79  -.704  2.17 | -.004  .002  -.096  .095  -.033  .097 | -.004  -.200  -2.08  1.65  -.780  2.08 | -.008  -.015  -.102  .087  -.037  .093 |
| Shift schedule^d^ |  |  |  |  |  |  | .583 | .049 |
| *∆R^2^* |  |  |  | .051 |  | .029 |  | .002 |
| *R^2^* |  | .003 |  | .053 |  | .082 |  | .084 |
| *∆F* |  | .324 |  | 6.21^*^ |  | 1.18 |  | .493 |
| *F model* |  | .324 |  | 3.27^*^ |  | 2.02^*^ |  | 1.88^*^ |

Unstandardized (B) and standardized (ꞵ) regression coefficients are shown.

Model 1: age and gender; Model 2: age, gender, BMI and physical activity; Model 3: age, gender, BMI, physical activity, years of shift work exposure and occupations; Model 4: age, gender, BMI, physical activity, years of shift work exposure, occupations and shift schedule. Values in **bold** indicate a statistically significant result.

^a^ 0 = male, 1 = female

^b^ Physical activity in MET minutes/week transformed into square root values

^c^“professional” occupational group used as reference

^d^ 0 = fixed night shift, 1 = rotating shift

^*^p < .05; ^**^p <.001

Table S4 Hierarchical regression predicting carbohydrate intake (%EI) in shift workers (n= 237)

|  | **Model 1** | | **Model 2** | | **Model 3** | | **Model 4** | |
| --- | --- | --- | --- | --- | --- | --- | --- | --- |
| **Variable** | **B** | **ꞵ** | **B** | **ꞵ** | **B** | **ꞵ** | **B** | **ꞵ** |
| Constant  Age  Gender^a^ | 44.5^**^  **-.089**  **2.14^*^** | -.120  **.148^*^** | 47.6^**^  **-.101^*^**  **2.25^*^** | **-.135^*^**  **.156^*^** | 49.8^**^  -.094  1.53 | -.126  .107 | 50.3^**^  -.099  1.45 | -.133  .100 |
| BMI  Physical activity^b^ |  |  | -.099  .010 | -.081  .050 | -.107  .014 | -.090  .055 | -.109  .014 | -.089  .073 |
| Shift work exposure  Community/personal service^c^  Technicians/trades^c^  Managers/clerical/sales^c^  Machinery operators/ drivers^c^  Labourers^c^ |  |  |  |  | -.053  -1.96  -1.93  -1.41  -1.21  **-4.55^*^** | -.075  -.119  -.073  -.063  -.044  **-.166^*^** | -.051  -1.76  -1.82  -1.29  -1.14  **-4.48^*^** | -.075  -.110  -.073  -.056  -.044  **-.165^*^** |
| Shift schedule^d^ |  |  |  |  |  |  | -.534 | -.037 |
| *∆R^2^* |  |  |  | .010 |  | .027 |  | .001 |
| *R^2^* |  | .036 |  | .045 |  | .072 |  | .074 |
| *∆F* |  | 4.32^*^ |  | 1.20 |  | 1.10 |  | .276 |
| *F model* |  | 4.32^*^ |  | 2.76^*^ |  | 1.76 |  | 1.62 |

Unstandardized (B) and standardized (ꞵ) regression coefficients are shown.

Model 1: age and gender; Model 2: age, gender, BMI and physical activity; Model 3: age, gender, BMI, physical activity, years of shift work exposure and occupations; Model 4: age, gender, BMI, physical activity, years of shift work exposure, occupations and shift schedule. Values in **bold** indicate a statistically significant result.

^a^ 0 = male, 1 = female

^b^ Physical activity in MET minutes/week transformed into square root values

^c^“professional” occupational group used as reference

^d^ 0 = fixed night shift, 1 = rotating shift

^*^p < .05; ^**^p <.001

Table S5 Hierarchical regression predicting saturated fat (%EI) in shift workers (n= 237)

|  | **Model 1** | | **Model 2** | | **Model 3** | | **Model 4** | |
| --- | --- | --- | --- | --- | --- | --- | --- | --- |
| **Variable** | **B** | **ꞵ** | **B** | **ꞵ** | **B** | **ꞵ** | **B** | **ꞵ** |
| Constant  Age  Gender^a^ | 13.8^**^  -.005  .002 | -.017  .000 | 12.4^**^  .007  -.118 | .021  -.019 | 12.4^**^  .016  -.458 | .051  -.074 | 12.3^**^  .017  -.441 | .054  -.072 |
| BMI  Physical activity^b^ |  |  | .061  **-.015^*^** | .118  **-.183^*^** | .047  **-.013^*^** | .091  **-.165^*^** | .048  **-.013^*^** | .092  **-.165^*^** |
| Shift work exposure  Community/personal service^c^  Technicians/trades^c^  Managers/clerical/sales^c^  Machinery operators/ drivers^c^  Labourers^c^ |  |  |  |  | -.001  .273  -.853  1.31  -1.35  .958 | -.004  .040  -.081  .133  -.122  .083 | -.002  .230  -.878  1.28  -1.37  .942 | -.006  .034  -.083  .130  -.124  .081 |
| Shift schedule^d^ |  |  |  |  |  |  | .115 | .019 |
| *∆R^2^* |  |  |  | .051 |  | .046 |  | .000 |
| *R^2^* |  | .000 |  | .051 |  | .097 |  | .098 |
| *∆F* |  | .032 |  | 6.20^*^ |  | 1.93 |  | .072 |
| *F model* |  | .032 |  | 3.11^*^ |  | 2.44^*^ |  | 2.21^*^ |

Unstandardized (B) and standardized (ꞵ) regression coefficients are shown.

Model 1: age and gender; Model 2: age, gender, BMI and physical activity; Model 3: age, gender, BMI, physical activity, years of shift work exposure and occupations; Model 4: age, gender, BMI, physical activity, years of shift work exposure, occupations and shift schedule. Values in **bold** indicate a statistically significant result.

^a^ 0 = male, 1 = female

^b^ Physical activity in MET minutes/week transformed into square root values

^c^“professional” occupational group used as reference

^d^ 0 = fixed night shift, 1 = rotating shift

^*^p < .05; ^**^p <.001

Table S6 Hierarchical regression predicting added sugar intake (%EI) in shift workers (n= 237)

|  | **Model 1** | | **Model 2** | | **Model 3** | | **Model 4** | |
| --- | --- | --- | --- | --- | --- | --- | --- | --- |
| **Variable** | **B** | **ꞵ** | **B** | **ꞵ** | **B** | **ꞵ** | **B** | **ꞵ** |
| Constant  Age  Gender^a^ | 12.0^**^  **-.098^*^**  .569 | **-.180^*^**  .054 | 15.3^**^  **-.097^*^**  .549 | **-.179^*^**  .052 | 13.6^**^  -.066  1.06 | -.122  .101 | 13.9^**^  -.069  1.01 | -.127  .096 |
| BMI  Physical activity^b^ |  |  | -.074  -.010 | -.083  -.073 | -.078  -.010 | -.088  -.073 | -.080  -.010 | -.089  -.074 |
| Shift work exposure  Community/personal service^c^  Technicians/trades^c^  Managers/clerical/sales^c^  Machinery operators/ drivers^c^  Labourers^c^ |  |  |  |  | -.038  1.67  1.71  1.52  .613  -.442 | -.077  .143  .095  .090  .033  -.022 | -.037  1.79  1.78  1.60  .655  -.397 | -.075  .153  .098  .095  .035  -.020 |
| Shift schedule^d^ |  |  |  |  |  |  | -.318 | -.030 |
| *∆R^2^* |  |  |  | .011 |  | .029 |  | .001 |
| *R^2^* |  | .035 |  | .046 |  | .075 |  | .075 |
| *∆F* |  | 4.24* |  | 1.31 |  | 1.17 |  | .185 |
| *F model* |  | 4.24* |  | 2.78^*^ |  | 1.82 |  | 1.67 |

Unstandardized (B) and standardized (ꞵ) regression coefficients are shown.

Model 1: age and gender; Model 2: age, gender, BMI and physical activity; Model 3: age, gender, BMI, physical activity, years of shift work exposure and occupations; Model 4: age, gender, BMI, physical activity, years of shift work exposure, occupations and shift schedule. Values in **bold** indicate a statistically significant result.

^a^ 0 = male, 1 = female

^b^ Physical activity in MET minutes/week transformed into square root values

^c^“professional” occupational group used as reference

^d^ 0 = fixed night shift, 1 = rotating shift

^*^p < .05; ^**^p <.001
